# FGD1 promotes tumor progression and regulates tumor immune response in osteosarcoma via inhibiting PTEN activity

**DOI:** 10.7150/thno.41279

**Published:** 2020-02-03

**Authors:** Wei Wu, Doudou Jing, Zibo Meng, Binwu Hu, Binlong Zhong, Xiangyu Deng, Xin Jin, Zengwu Shao

**Affiliations:** 1 Department of Orthopedics, Union Hospital, Tongji Medical College, Huazhong University of Science and Technology, Wuhan, 430022, China; 2 Cancer center, Union Hospital, Tongji Medical College, Huazhong University of Science and Technology, Wuhan, 430022, China

**Keywords:** FGD1, PTEN, osteosarcoma, tumor progression, PD-L1

## Abstract

**Rationale**: Mesenchymal cell-derived osteosarcoma is a rare malignant bone tumor affecting children and adolescents. PTEN down-regulation or function-loss mutation is associated with the aggressive of osteosarcoma. Explicating the regulatory mechanism of PTEN might highlight new targets for improving the survival rate of osteosarcoma patients.

**Methods**: The clinical relevance of FGD1 was examined by the TCGA data set, Western blotting and immunohistochemistry of osteosarcoma microarray slides. Functional assays, such as the MTS assay, colony formation assay and xenografts, were used to determine the biological role of FGD1 in osteosarcoma. The protein-protein interaction between FGD1 and PTEN was detected via co-immunoprecipitation. The relationship between FGD1 and PD-L1 was examined by Western blot analysis, RT-qPCR and immunohistochemistry.

**Results**: In this study, analysis of the TCGA data set of sarcomas revealed that FGD1 was over-expressed with the highest P values. Then, we demonstrated that FGD1 was also abnormally up-regulated in osteosarcoma with unfavorable prognosis. Aberrant expressed FGD1 promoted the osteosarcoma tumor cell proliferation and invasion. Moreover, we found that FGD1 was participated in activating PI3K/AKT signaling pathway by interacting with PTEN. Finally, we showed that FGD1 was capable of regulating the tumor immune response via the PTEN/PD-L1 axis in osteosarcoma.

**Conclusions**: Our data suggested that abnormally over-expressed FGD1 functions as an oncogenic protein to promote osteosarcoma progression through inhibiting PTEN activity and activating PI3K/AKT signaling. Notably, FGD1 increased PD-L1 expression in a PTEN dependent manner and modulated the sensitivity of immune checkpoint-based immunotherapy in osteosarcoma. Thus, FGD1 might be a potential target for improving the survival rate of osteosarcomas.

## Introduction

Mesenchymal cell-derived osteosarcoma is a rare malignant bone tumor affecting children and adolescents 1. In the past decades, surgical resection and traditional adjuvant chemotherapy were applied to treat osteosarcoma. Consequently, the 5-year survival rate of high-grade osteosarcoma reached more than 70% 2. Low-grade osteosarcoma is characterized by early metastasis and easy recurrence, which profoundly shortens the patient's survival time 2, 3. About 20% of osteosarcoma cases are diagnosed with metastasis at the first visit 1. It is worth noting that osteosarcoma patients with distal metastasis or local relapse are resistant to conventional chemotherapy. Despite the use of intensive chemotherapy to treat these osteosarcoma patients, chemotherapy drugs did not change the survival time of patients except for serious side effects 1. Therefore, exploring and developing new effective therapeutic methods are an urgent need for osteosarcoma patients.

Recently, a high level of genomic instability was defined in osteosarcoma 1 and several oncogenic pathways, such as the phosphatidylinositol 3-kinase (PI3K)/AKT signaling pathway, were shown to contribute towards the tumorigenesis of osteosarcoma 4. The PI3K/AKT pathway is activated in almost all advanced osteosarcoma cases 4. A cascade of events of the PI3K/AKT pathway is involved in modulating the osteosarcoma tumor cell proliferation, cell cycle, tumor cell apoptosis, migration, invasion and chemotherapy resistance 4. Recently, small molecules targeting AKT or downstream proteins, including mTOR, have shown a promising anti-tumor effect on osteosarcoma cells 4 and in clinical trials 5. Thus, a better understanding of the pathogenesis of the PI3K/AKT pathway might reveal more candidates for osteosarcoma treatment.

Phosphatase and tensin homologue (*PTEN*) gene is located on chromosome 10 and encodes a protein containing 403 amino acids 6, 7. PTEN consists of an N-terminal phosphatase domain involved in modulating lipid phosphatase activity, and a C-terminal domain responsible for PTEN degradation 8. PTEN functions as a tumor suppressor by dephosphorylating phosphatidylinositol (3,4,5)-triphosphate (PtdIns-3,4,5-P3) and negatively regulating the PI3K/AKT pathway 9. PTEN is reported to inhibit the tumor growth and invasion in U-2OS and MG63 osteosarcoma cell lines 10. PTEN down-regulation or loss-of-function mutation is found in osteosarcoma and has a close relationship with the aggressiveness of osteosarcoma 10. Explicating the regulatory mechanism of PTEN could highlight new targets for improving the survival rate of osteosarcoma patients. In this study, we identified FYVE, RhoGEF and PH domain-containing protein 1 (FGD1) as a bona fide binding partner of PTEN. Our results revealed that FGD1 was markedly overexpressed and might be a prognostic biomarker in osteosarcoma patient specimens. Moreover, our data indicated that FGD1 promoted osteosarcoma progression by increasing the proliferation and invasion ability of the tumor cells. Interestingly, we found that FGD1 could bind with PTEN to inhibit the activity of PTEN and activate PI3K/AKT/NK-kB signaling in osteosarcoma cells. Finally, we demonstrated that FGD1 could regulate osteosarcoma immune response through PTEN/PD-L1 axis. Thus, FGD1 might be a novel candidate for osteosarcoma therapy.

## Material and Methods

### Cell lines and cell culture

We purchased four human osteosarcoma cell lines (MNNG/HOS, MG-63, 143B and U-2OS) and a mouse osteosarcoma cell line (K7M2-WT) from the Cell Bank of China Academy of Sciences (Shanghai, China). MNNG/ HOS, MG-63 and U-2OS cells were cultured in α-MEM (HyClone, USA) supplemented with 10% fetal bovine serum (Gibco, USA). The 143B cells were cultured in McCoy's 5A medium (Gibco, USA) supplemented with 10% fetal bovine serum. K7M2-WT cells were cultured in DMEM, High Glucose (Gibco, USA) supplemented with 10% fetal bovine serum. All the cell lines were maintained at 37°C with 5% CO_2_ in a humidified incubator.

### Plasmids, antibodies and chemicals

We purchased mammalian expression vectors for Flag-FGD1 and Myc-PTEN recombinant proteins from GeneChem (Shanghai, China). The FGD1 antibody (#PA5-40416) was purchased from Thermo Fisher Scientific (working dilution 1:500); GAPDH (#ab9485) was from Abcam (working dilution 1:5000); PTEN (#9188S) was from Cell Signaling Technology (working dilution 1:1000); AKT (#4691T) was from Cell Signaling Technology (working dilution 1:1000); Phospho-AKT (Ser473) (#4060S) was from Cell Signaling Technology (working dilution 1:1000); FOXO1 (#2880S) was from Cell Signaling Technology (working dilution 1:1000); Phospho-FOXO1 (Ser319) (#2486) was from Cell Signaling Technology (working dilution 1:1000); PD-L1 (#13684S) was from Cell Signaling Technology (working dilution 1:1000); Flag (#A5712) was from Bimake (working dilution 1:2000). MK2206 and Ly294002 were purchased from MedChemExpress (Shanghai, China).

### Western blotting of cells and tissues

The rationality of using human tissue (12 pairs of matched osteosarcoma/adjacent non-tumor tissues) was audited by the Local Ethics Committee (Tongji Medical College, China). All patients or their guardians provided written informed consent. Tumor tissues (after grinding) or cells were lysed with RIPA lysis buffer (Beyotime, China) containing 1% phosphatase and protease inhibitors. The protein content in the lysates was determined using the BCA protein assay kit (Beyotime Biotechnology, China). Proteins were separated by sodium dodecyl sulphate-polyacrylamide gel electrophoresis (SDS-PAGE) and transferred to PVDF membranes (Millipore, Massachusetts, USA). 5% skim milk was used to block the PVDF membranes for 1 h at room temperature, followed by incubation with specific antibody overnight at 4°C. Next, the membrane was washed with 1X TBST and incubated with secondary antibodies for 1 h at room temperature. In the end, the immunoreactive bands were exposed by using the ECL Kit (Thermo Fisher Scientific) under the illumination of X-rays.

### Flow cytometry analysis

We divided the osteosarcoma cells into four treatment groups (shControl, shControl+MK2206, shFGD1 and shFGD1+MK2206). All the cells were harvested and washed with 1X PBS. Then, we centrifuged the cells and discarded the liquid supernatant. We used a binding buffer to resuspend the cells before being labelled by Annexin V-FITC/PI double-staining (Keygen Biotech, China) in the dark. All the samples were analyzed by flow cytometry. The U-2OS, MNNG/HOS and 143B cells were infected by shControl or shFGD1. The cells were harvested and washed with 1X PBS followed by fixing in 4% paraformaldehyde for 15 min. After washing with 1X PBS, cells were incubated with chilled 100% methanol for 30 min on ice. We washed the cells with PBS again and incubated the cells with PD-L1 antibody (BioLegend, APC anti-human CD274, clone 29E.2A3) or isotype IgG (BioLegend, APC anti-human IgG Fc Antibody, clone HP6017) for 15 min at room temperature. Subsequently, the cells were given three washes with PBS and suspended in 1X PBS to be analyzed by flow cytometry.

### *In vivo* assay

All the animal experimental protocols were authorized by the Ethics Committee of Tongji Medical College, Huazhong University of Science and Technology. Nude mice (BALB/c, female, 4 to 5-week-old, 18-20 g) were injected hypodermically with 5×10^6^ MNNG/HOS cells. All mice were randomly divided into three groups (n=5/group) and the cells for injecttion were treated differently (Control, shFGD1 and shFGD1+Tsin-Flag-FGD1) or (shControl, shFGD1, shControl+MK2206 and shFGD1+MK2206). The shFGD1 and shControl were purchased from RiboBio (Guangzhou, China). The mice were administered normal saline solution or MK2206 (120/mg/kg/d), intraperitoneally. Tumor volumes were calculated from the length and the width using the following formula: volume (mm^3^) = L x W ^2^/2. Three weeks after injection, the animals were euthanized and tumors were harvested, weighed, and fixed in 4% paraformaldehyde.

### Phosphatidylinositol-3,4,5-trisphosphate (PIP3) phosphatase assay

PIP3 phosphatase assay was performed following the manufacturer's protocol of Assay kit for quantitative determination of PTEN activity by colorimetry (GMS50064.1, Genmed, Shanghai). Briefly, 5X10^6^ pancreatic cancer cells were harvested from 6-well plated and lysed by specific lysis buffer (Reagent B). Then, the sample were reacted with Reagent E buffer at 37°C for 10 min. The reaction was stopped by Reagent F buffer. Reagent G buffer for color rendering was added and incubated in the room temperature in the dark for 15min. PIP3 was dephosphorylated by PTEN to release free phosphate, which reacted with malachite green dye and measured by spectrophotometer at 660nm.

### Statistical analysis

Statistical analyses were performed with one-sided or two-sided paired Student's t-test for single comparison and one-way ANOVA with a post hoc test for multiple comparisons. P value < 0.05 was considered statistically significant. All the values are expressed as the mean ± SD.

Other methods are provided in Supplementary information.

## Results

### Expression of FGD1 is up-regulated in osteosarcoma patient specimens and associated with poor prognosis

**Figure 1:**
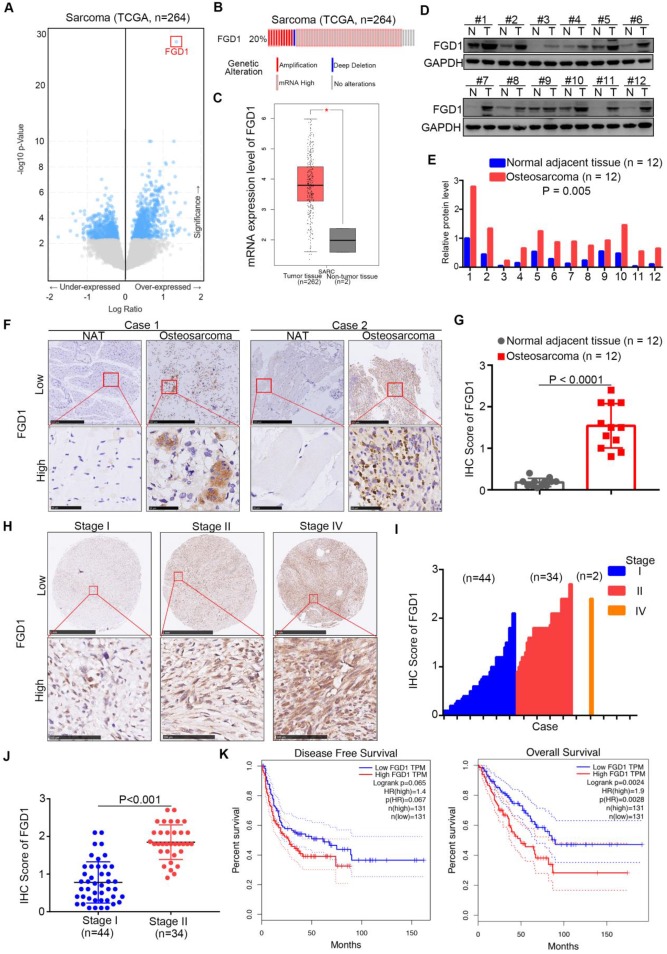
** Expression of FGD1 is up-regulated in osteosarcoma patient specimens and associated with poor prognosis. A,** Bioinformatics analysis of the TCGA data set by using the cbioportal web tool (http://www.cbioportal.org/) to get the under-/over-expressed genes in sarcoma. **B**, the mRNA expression levels of FGD1 in sarcoma. **C**, analysis of the mRNA expression levels of FGD1 by using the GEPIA web tool (http://gepia.cancer-pku.cn/). **D**-**G**, the protein levels of FGD1 from osteosarcoma specimens and corresponding adjacent non-tumor tissue (NAT) (n = 12) were detected by Western blotting analysis (D and E) and IHC analysis (F and G). P values as indicated. **H**-**J,** the protein levels of FGD1 from osteosarcoma tissue microarray (osteosarcoma specimens n = 80) were determined by IHC analysis. The typical image of FGD1 in different stage as indicated in H.** K**, the disease-free survival and overall survival rate in low/high FGD1 group was analyzed by the GEPIA web tool.

First, we analyzed the TCGA data set of sarcomas to explore the up-/down-regulated genes, which might be therapeutic targets for sarcoma patients 11, 12. Interestingly, we observed that FGD1 was over-expressed in the data set with the highest P values (Figure 1A). It has been found that the mRNA levels of FGD1 were up-regulated in 20 percent of sarcoma patients (Figure 1B). Moreover, the mRNA expression levels of FGD1 in normal tissues were found to be lower than those in the sarcoma tissues using GEPIA or Oncomine web tools (Figure 1C and Figure S1A) 13. Similarly, the protein expression of FGD1 in osteosarcoma tissues was higher than that in the adjacent normal tissues after Western blot analysis or immunohistochemistry (IHC) staining of patient samples (Figure 1D-G), which is consistent with the mRNA levels (Figure S1B). Furthermore, we stained FGD1 in the osteosarcoma tissue microarray (osteosarcoma specimens n = 80) and found that FGD1 expression was positively correlated with the tumor stage (Figure 1H-1J). At last, the survival assay performed using the GEPIA web tool indicated that over-expression of FGD1 shortened the disease-free survival time (Logrank P = 0.065) and overall survival time (Logrank P = 0.0024) of osteosarcoma patients (Figure 1K). Besides, FGD1 was also overexpressed in melanoma and had a close relationship with the prognosis in melanoma patients (Figure S1C-S1D). Together, the results demonstrate that FGD1 is up-regulated in osteosarcoma and could be used as a biomarker to predict the prognosis of osteosarcoma in patients.

### Abnormally overexpressed FGD1 results in osteosarcoma progression

**Figure 2:**
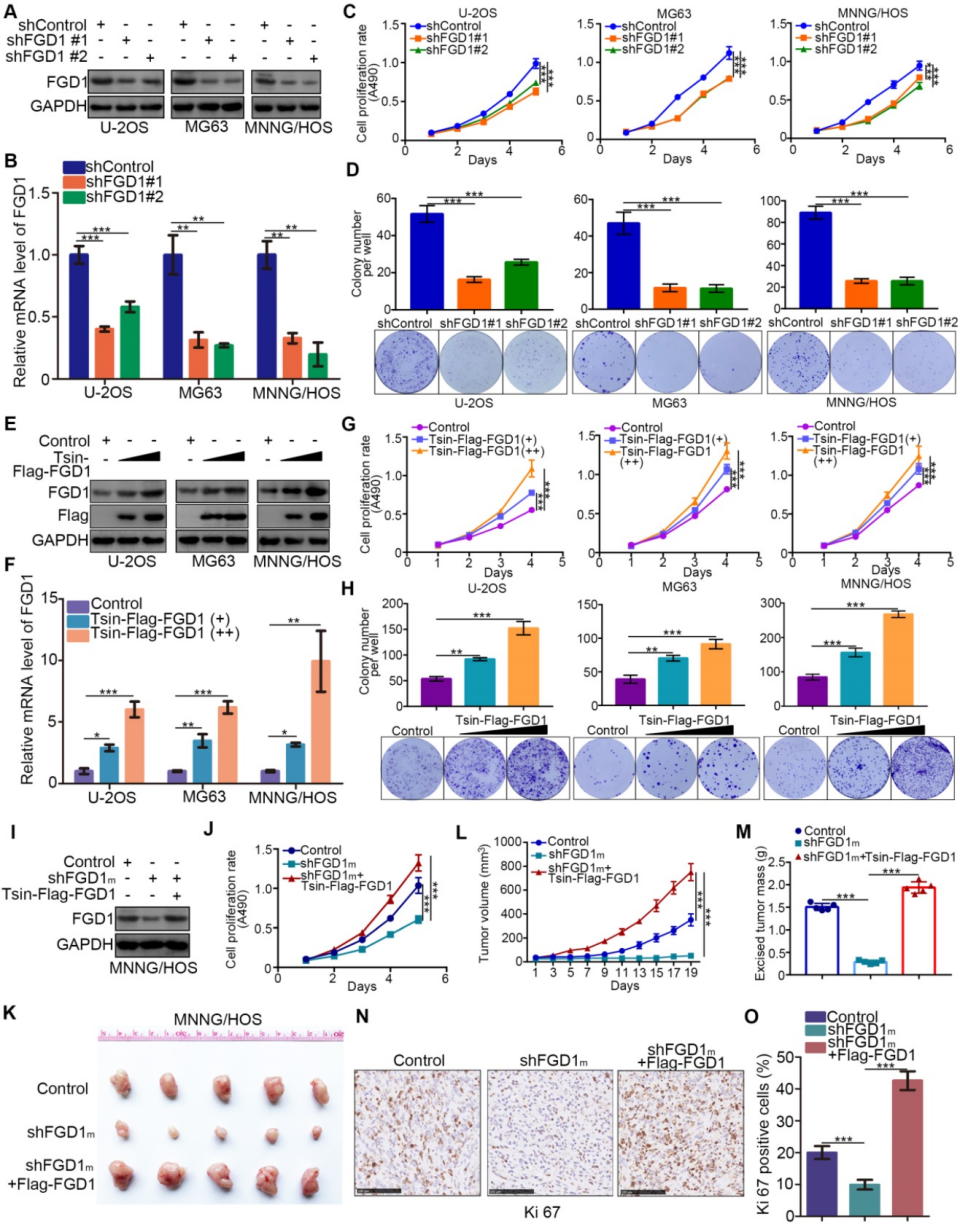
** Abnormally overexpressed FGD1 results in osteosarcoma progression. A-D,** the osteosarcoma tumor cells (U-2OS, MG63 and MNNG/HOS) were infected with indicated shRNAs. After 72 h, cells were harvested for Western blotting analysis (A), RT-qPCR analysis (B), MTS cell proliferation assay (C) and colony formation assay (D). Data presented as Mean ± SD with three replicates. **, P < 0.01; ***, P < 0.001. **E**-**H**, U-2OS, MG63 and MNNG/HOS cells were transfected with indicated plasmids for 72h. Cells were used for Western blotting analysis (E), RT-qPCR analysis (F), MTS cell proliferation assay (G) and colony formation assay (H). Data presented as Mean ± SD with three replicates. *, P < 0.05; **, P < 0.01; ***, P < 0.001.** I-O**, MNNG/HOS cells were transfected with indicated constructs. After 72 h transfection and puromycine selection, cells were harvested for Western blotting analysis (I), MTS assay (J), or injected subcutaneously into the nude mice for xenografts assay (K). The tumor growth curve (L) and excised tumor mass (M) as indicated. The tumor was subjected to Ki-67 staining (N and O). ***, P < 0.001.

Given that FGD1 might be a prognostic biomarker of osteosarcoma, understanding the role of FGD1 in osteosarcoma tumor cells could verify its oncogenic property. FGD1 was repressed through infection with gene-specific short-hairpin RNA in U-2OS, MG63 and MNNG/HOS cells, respectively (Figure 2A and 2B). FGD1 inhibition profoundly decreased the osteosarcoma tumor cell proliferation ability *in vitro*, as revealed by the MTS assay and colony formation assay (Figure 2C and 2D). On the contrary, FGD1 overexpression after transfection with FGD1 plasmids resulted in an increasing tumor cell growth in osteosarcoma cells (Figure 2E-2H). Moreover, we observed that recusing the expression of FGD1 could reverse the decreased tumor cell proliferation effect induced by repression of FGD1 in MNNG/HOS cells (Figure 2I and 2J). Furthermore, the xenograft tumor assay was used to evaluate the capability of tumor cell growth *in vivo*. Similarly, knockdown of FGD1 suppressed the tumor growth and decreased the number of Ki-67-positive cells, but overexpression of FGD1 reversed this process (Figure 2K-2O). Meanwhile, we also found that knockdown of FGD1 blocked the tumor cell migration and regulated the cell cycle in osteosarcoma cells (Figure S2A-S2C). Collectively, our data suggest that FGD1 promotes the osteosarcoma tumor cell progression *in vivo* and *in vitro*.

### Knockdown of FGD1 represses activation of PI3K/AKT signaling in osteosarcoma cells

**Figure 3:**
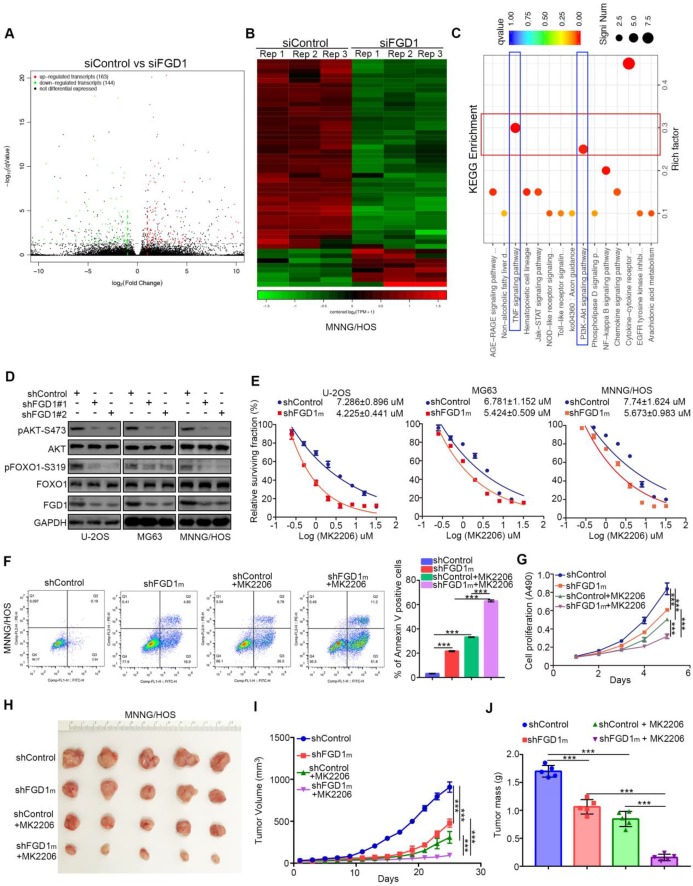
** Knockdown of FGD1 represses activation of PI3K/AKT signaling in osteosarcoma cells. A-C,** MNNG/HOS cells were transfected with indicated constructs for 48h. Cells were subjected to RNA-seq analysis (A and B) and subsequent KEGG pathway enrichment (C). **D**, The whole cell lysates (WCL) of U-2OS, MG63 and MNNG/HOS cells after transfected with indicated constructs for 72 h. **E**, U-2OS, MG63 and MNNG/HOS cells were subjected to measuring the IC50 values of MK2206 after transfected with indicated shRNAs (shFGD1m means mixed with two different shRNA). The IC50 values as indicated.** F-J**, MNNG/HOS cells were infected with indicated constructs. After 72 h infection, cells were harvested for FACS assay (F) and MTS assay (G). Then, MNNG/HOS cells were subcutaneously into the nude mice for xenografts assay (H). The tumor growth curve (I) and the excise tumor mass (J) as indicated. ***, P < 0.001.

As FGD1 is responsible for the osteosarcoma tumor cell progression, the mechanism underlying the modulation of this process by FGD1 needs to be studied further. The RNA-Seq data analysis was conducted in MNNG/HOS cells with or without FGD1 knockdown (Figure 3A and 3B). Subsets of the down-/up-regulated genes by silencing FGD1 in three-replicates were identified (Figure 3A and 3B). GO and KEGG enrichment analysis indicated that down-regulated genes overlapped with the PI3K/ AKT signaling pathway and TNF signaling pathway after repression of FGD1 in MNNG/HOS cells (Figure 3C, Figure S3A and S3B). To verify the above finding, we performed knockdown of FGD1 in three osteosarcoma cell lines: U-2OS, MG63 and MNNG/HOS. FGD1 silencing inhibited the phosphorylation of AKT and FOXO1, which is a well-known downstream event of the PI3K/AKT pathway (Figure 3D). Besides, knockdown of FGD1 led to the osteosarcoma tumor cells being more sensitive to pan-AKT inhibitors (MK2206), as depicted by the decreasing IC50 values of MK2206 in three cell lines (Figure 3E). In contrast, ectopically overexpressed FGD1 led to an increase in the IC50 values of MK2206 in three osteosarcoma tumor cell lines (Figure S4A). Moreover, FGD1 repression not only resulted in apoptotic cell death but also enhanced the growth-inhibitory effect of MK2206 in osteosarcoma tumor cell lines *in vivo* and *in vitro* (Figure 3F-3J, Figure S4B and S4C), which indicates that the PI3K/AKT pathway plays an important role in mediating the biological function of FGD1 in osteosarcoma cells.

### FGD1 interacts with PTEN to inhibit PTEN phosphatase activity in osteosarcoma cells

**Figure 4:**
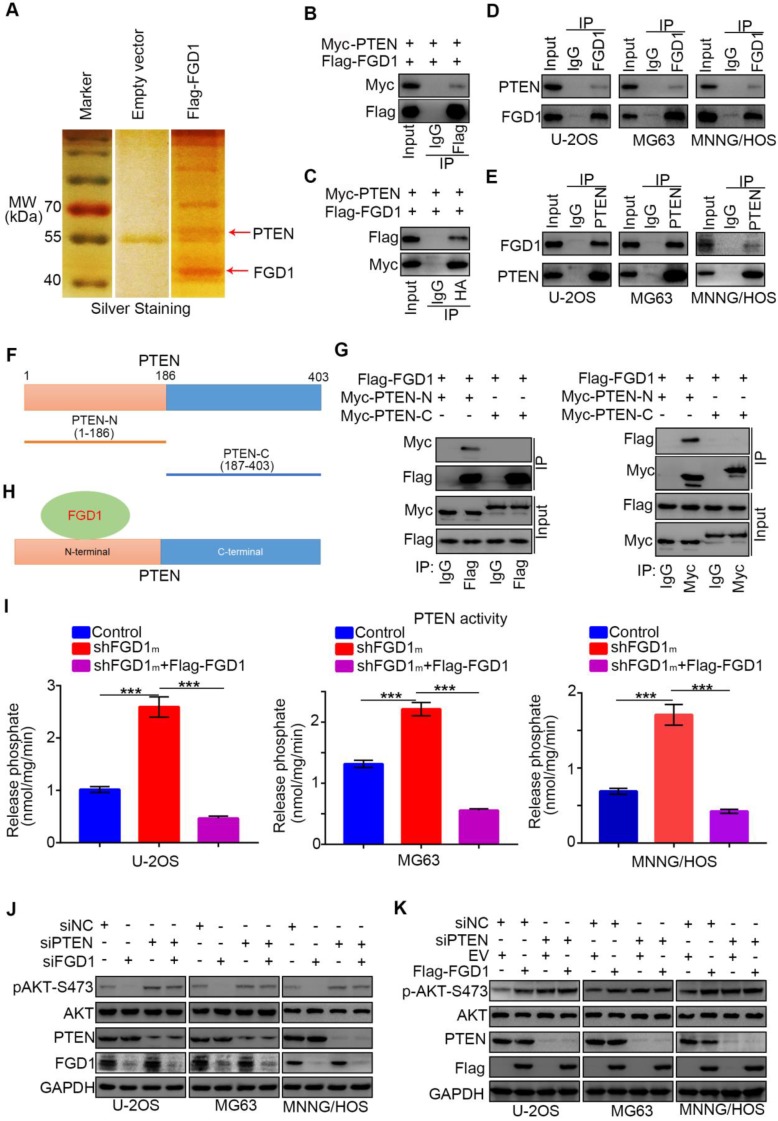
** FGD1 interacts with PTEN to inhibit PTEN phosphates activity in osteosarcoma cells. A,** the WCL of 293T cells were subjected to silver staining and mass spectrometry after transfected indicated plasmids. **B-C**, 293T cells transfected with indicated plasmids was harvested for co-immunoprecipitation. **D-E**, Western blotting analysis of WCL of U-2OS, MG63 and MNNG/HOS cells. **F**, a schematic diagram depicting the domain of PTEN.** G**, Flag-FGD1, Myc-PTEN-C and Myc-PTEN-N were translated *in vitro*, and the co-immunoprecipitation was performed to evaluate the interaction between the PTEN recombination protein and FGD1. **H**, a schematic diagram depicting FGD1 interacted with the N-terminal region of PTEN.** I**, U-2OS, MG63 and MNNG/HOS cells were transfected with indicated constructs. After 72 h, the spend medium of each treatment group were collected for release phosphatase assay. ***, P < 0.001. **J**, Western blotting analysis of the WCL of osteosarcoma tumor cells (U-2OS, MG63 and MNNG/HOS) after transfected with indicated siRNAs. **K**, Western blotting analysis of the WCL of osteosarcoma tumor cells (U-2OS, MG63 and MNNG/HOS) after transfected with indicated plasmids.

Since we identified that FGD1 positively regulated the activation of the PI3K/AKT signaling pathway in osteosarcoma cells, the specific mechanism was still not explicit. To explore the underlying mechanism, Flag-tagged FGD1 constructs were transfected into 293T cells. Cell extracts were immunoprecipitated with IgG or Flag antibody and subjected to mass spectrometry analysis. Importantly, mass spectrometry analysis suggested that PTEN might be one of the binding partners of FGD1 (Figure 4A). As PTEN negatively modulated the PI3K/AKT pathway, we were interested in determining whether PTEN was the bridge between FGD1 and the PI3K/AKT pathway. Firstly, co-immunoprecipitation (Co-IP) was performed to verify the interaction between exogenously expressed Flag-FGD1 and Myc-PTEN in 293T cells (Figure 4B and 4C). Then, the binding between endogenous FGD1 and PTEN was confirmed in U-2OS, MG63 and MNNG/HOS cells (Figure 4D and 4E). To further identify the region of PTEN that interacted with FGD1, the PTEN-N (amino acids 1-186) and PTEN-C (amino acids 187-403) plasmids were constructed (Figure 4F). The recombinant proteins of Myc-PTEN-N, Myc-PTEN-C and Flag-FGD1 were expressed by the TNT Quick translation system 14. The *in vitro* Co-IP assay demonstrated that the N-terminal of PTEN interacted with FGD1 in cells (Figure 4G and 4H). Given that the N-terminal region of PTEN is essential for controlling the cytoplasmic localization and activity of PTEN 15, 16 and that PTEN and FGD1 were mutually exclusively in sarcoma patient samples (Figure S5A), we wondered whether FGD1 inhibits the function of PTEN in osteosarcoma cells. PIP3 phosphatase assay indicated that the knockdown of FGD1 increased the activity of PTEN and this effect could be diminished after rescuing the expression of FGD1 in U-2OS, MG63 and MNNG/HOS cell lines (Figure 4I). Moreover, we found that FGD1 silencing or overexpression-induced decrease or increase in the phosphorylation levels of AKT could be attenuated by knockdown of PTEN in osteosarcoma tumor cells (Figure 4J and 4K). Furthermore, our data demonstrated that PTEN knockdown diminishes the growth-inhibitory effect and rescues the down-regulated genes, such as* IL-6, PDGF8, CXCL1* and *CXCL8* 17, 18, after silencing FGD1 in osteosarcoma tumor cells (Figure S5C and S5D). Thus, our results suggest that FGD1 inhibit the function of PTEN through binding to the N-terminal region of PTEN.

### FGD1 increases PD-L1 expression in osteosarcoma cells

**Figure 5:**
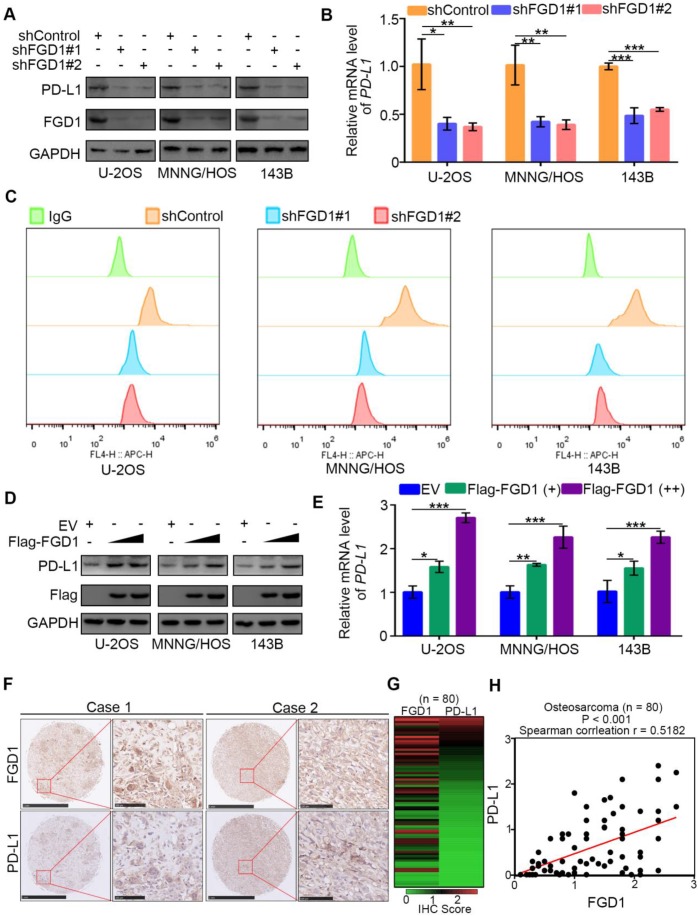
** FGD1 increases PD-L1 expression in OS cells. A-C,** U-2OS, MNNG/HOS and 143b cells were infected with indicated shRNAs. After 72 h, cells were harvested for Western blotting analysis (A), RT-qPCR analysis (B) and flow cytometry assay (C). Date showed as Mean ± SD with three replicates. *, P < 0.05; **, P < 0.01; ***, P < 0.001. **D-E**, U-2OS, MNNG/HOS and 143b cells were infected with indicated plasmids. After 48 h, cells were harvested for Western blotting analysis (D), and RT-qPCR analysis (E). Date showed as Mean ± SD with three replicates. *, P < 0.05; **, P < 0.01; ***, P < 0.001. **F-H**, IHC analysis of FGD1 and PD-L1 in osteosarcoma tissue microarray. The typical image of IHC as presented in panel F, the IHC scores of FGD1 or PD-L1 in each osteosarcoma specimens as indicated in panel G, the correlation between FGD1 and PD-L1 as showed in panel H.

Immune checkpoint-based immunotherapy, including the programmed cell death receptor-1 (PD-1) and its ligand (PD-L1) along with CTLA-4, represents a novel therapeutic strategy for osteosarcoma patients 19. PD-L1 is expressed on tumor cell surface and targeting PD-L1 by corresponding antibodies could reactivate the T cell response and suppress the tumor growth 20. Thus, understanding the regulatory mechanism of PD-L1 is important for improving the therapeutic effect of immune checkpoint-based therapy in osteosarcoma. It has been reported that PD-L1 is regulated by the PI3K/AKT signaling pathway and TNF/NF-κB pathway 21, 22. PTEN is a negative regulator of the PI3K/AKT signaling pathway and TNF/NF-κB pathway and also represses the expression of PD-L1 in cells 23. We observed that FGD1 might participate in regulation of the PI3K/AKT signaling pathway and TNF/NF-κB pathway by inhibiting the function of PTEN (Figure 3 and 4). Therefore, we aimed to explore whether FGD1 modulates PD-L1 expression in osteosarcoma tumor cells. Strikingly, knockdown of FGD1 by gene-specific shRNA down-regulated PD-L1 expression in U-2OS, 143B and MNNG/HOS cells (Figure 5A-5C). In contrast, PD-L1 was increased after ectopic expression of FGD1 in osteosarcoma tumor cells (Figure 5D and 5E). Then, we stained FGD1 and PD-L1 in the osteosarcoma tissue microarray (n = 80) by IHC to investigate the correlation between FGD1 and PD-L1. The IHC images and the corresponding IHC score of FGD1 and PD-L1 are presented in Figure 5F and Figure 5H. Furthermore, FGD1 was found to be positively correlated with PD-L1 in the osteosarcoma tissue microarray (P < 0.001, Spearman correlation r = 0.5182) (Figure 5G). Consistently, the GEPIA web tool analysis demonstrated that FGD1 mRNA expression was also positively correlated with PD-L1 mRNA in various types of tumors, including sarcoma, prostate cancer, pancreatic cancer, esophagus cancer, bladder cancer and melanoma (Figure S6A). Taken together, our data indicate that FGD1 promotes the expression of PD-L1 in osteosarcoma.

### FGD1 regulates immune response through the PTEN/PD-L1 axis in osteosarcoma

**Figure 6:**
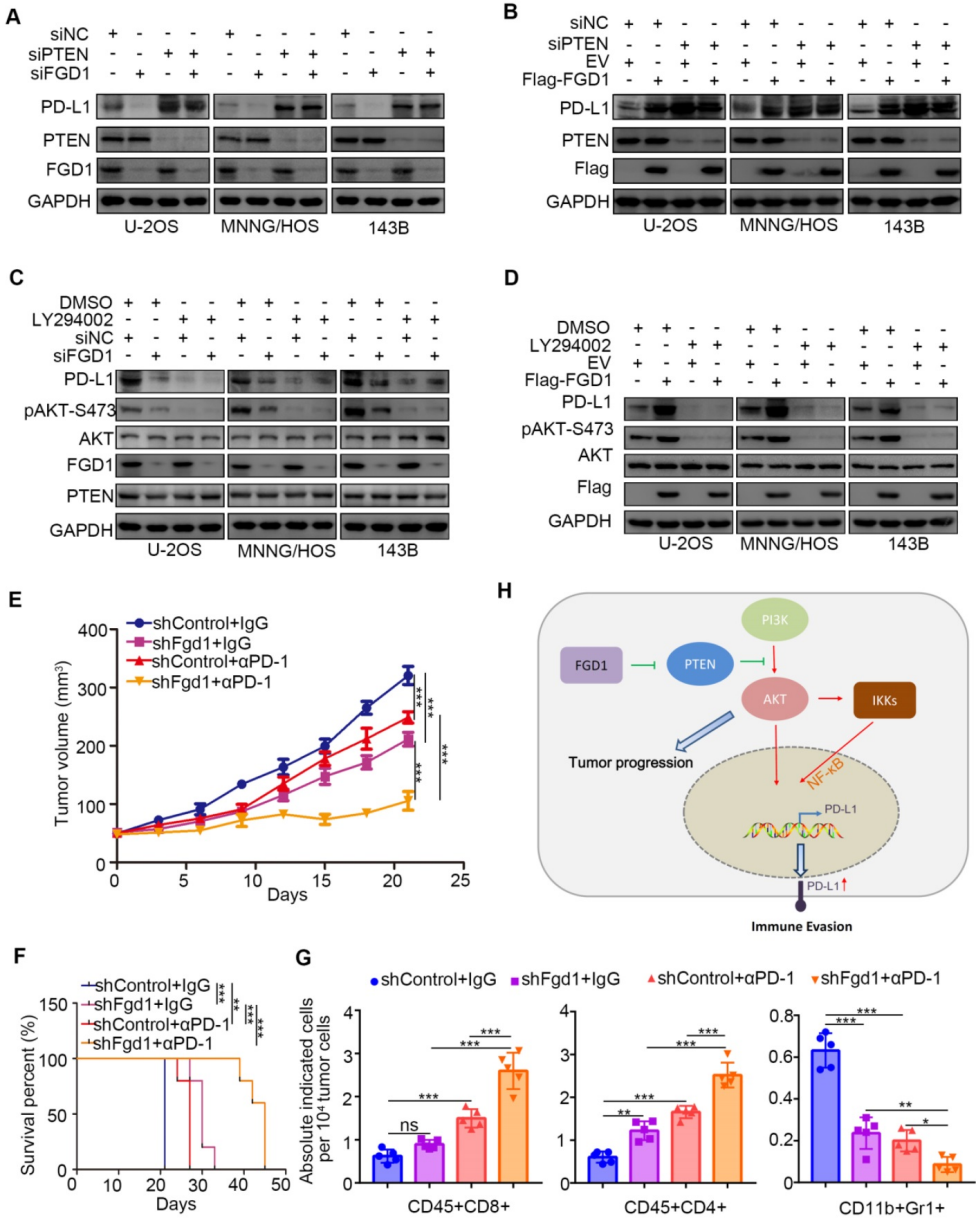
** FGD1 regulates OS immune response through PTEN/PD-L1 axis. A,** U-2OS, MNNG/HOS and 143B cells were infected with indicated siRNAs. After 48 h, cells were harvested for Western blotting analysis. **B**, U-2OS, MNNG/HOS and 143B cells were transfected with indicated constructs. 48 h post-transfection, cells were subjected to Western blotting analysis. **C**, U-2OS, MNNG/HOS and 143B cells were infected with indicated siRNAs. After 48 h, cells were treated with or without LY294002 (40 uM) for other 24 h. WCL of cells collected for Western blotting analysis. **D**, U-2OS, MNNG/HOS and 143B cells were infected with indicated constructs. After 48 h, cells were treated with or without LY294002 (40 uM) for other 24 h. WCL of cells collected for Western blotting analysis. **E-G**, K7M2 cells were infected with lentivirus vectors expressing control or Fgd1-specific shRNAs. 72 h after puromycin selection, 5 x 10^6^ cells were injected subcutaneously into C57BL/6 mice. Mice (n=5/group) were treated with anti-PD-L1 (200 µg) or non-specific IgG for 45 days. Growth curves of tumors with different treatments are shown in (E). Kaplan-Meier survival curves for each treatment group demonstrate the improved efficacy of combining PD-L1 mAb with the knockdown of Fgd1. **, P < 0.01; ***P < 0.001. (Gehan-Breslow-Wilcoxo test) (F). At the end of treatment, the numbers of infiltrated CD45^+^CD8^+^ T cells, CD45^+^CD4^+^ T cells, and CD11b^+^Gr1^+^ myeloid cells in tumors with different treatments were analyzed by FACS (G). All data are shown as mean values ± SD. ns, not significant, ** P < 0.01, *** P < 0.001. **H**, a hypothesis model depicting that FGD1 inhibits PTEN to trigger PI3K/AKT/NK-kB signaling pathway activation and promote tumor progression in osteosarcoma. Meanwhile, FGD1 modulated immune response of osteosarcoma via PTEN/PD-L1 axis.

To further investigate whether FGD1 regulates PD-L1 through PTEN, we performed knockdown of FGD1 and PTEN individually and in combination in the U-2OS, 143B and MNNG/HOS cells. PTEN knockdown up-regulated protein and mRNA levels of PD-L1 and most importantly attenuated the decreasing/increasing PD-L1 expression effect induced by FGD1 repression/overexpression in osteosarcoma tumor cells, respectively (Figure 6A and 6B, Figure S6B and S6C). Similarly, treatment with PI3K inhibitors suppressed the protein and mRNA levels of PD-L1 and diminished the decreasing/increasing PD-L1 expression effect produced by FGD1 repression/overexpression in osteosarcoma tumor cells, respectively (Figure 6C and 6D, Figure S6D and S6E). The above data suggest that PTEN is the key mediator for FGD1 induced PD-L1 expression in osteosarcoma cells. Next, we sought to find whether FGD1 modulated the immune response via PD-L1 in osteosarcoma. Murine osteosarcoma tumor cell line K7M2 infected with shControl or shFgd1 were subcutaneously injected into the right dorsal flank of immune-proficient mice. After the tumor reached the volume of 50 mm^3^, mice were subjected to IgG/PD-1 antibody administration indicated in figure S6F. The tumor growth curve was constructed every other day until the tumor volume went beyond 300 mm^3^ (Figure 6E). In agreement with the previous findings, knockdown of FGD1 hindered the tumor growth and prolonged the survival time of the tumor-bearing mice (Figure 6E and 6F). Meanwhile, PD-1 antibody treatment inhibited the tumor growth, increased the survival time of mice, and resulted in an increased CD45^+^CD4^+^ and CD45^+^CD8^+^ T cell infiltration or decreased CD11b^+^Gr1^+^ myeloid cell infiltration in tumors compared to that in the control group (Figure 6E-6G) 24. Importantly, the FGD1 knockdown group treated with PD-1 not only manifested the slowest growth rate and longest survival time (Figure 6E and 6F), but also further increased CD45^+^CD4^+^ and CD45^+^CD8^+^ T cell infiltration and decreased CD11b^+^Gr1^+^ myeloid cell infiltration in tumors (Figure 6E-6G). Collectively, we could demonstrate that FGD1 is capable of regulating the immune response via the PTEN/PD-L1 axis in osteosarcoma.

## Discussion

FGD1 consists of Dbl homology (DH) and pleckstrin homology (PH) domains, implying that FGD1 could bind with the Rho GTPase family 25. It has been well documented that FGD1 is a Rho GTPase cell division cycle 42 (CDC42) exchange factor in cells 25. FGD1 is responsible for G1 cell cycle progression, podosome assembly, filopodia formation, and JNK mitogen-activated protein kinase in a CDC42-dependent manner 26, 27. FGD1 loss-of-function mutation results in Aarskog-Scott syndrome, including facial, skeletal and urogenital anomalies 27. FGD1 contributes to extracellular matrix formation 26, which is essential for tumor formation and bone development. However, the pathological impact of FGD1 in bone tumor, such as osteosarcoma, is still unclear. Here, we revealed that abnormally overexpressed FGD1 promoted osteosarcoma progression (Figure 1 and 2). The study further uncovered that the knockdown of FGD1 repressed the PI3K/AKT signaling pathway and NF-κB signaling pathway in a PTEN-dependent manner. Thus, our data indicated a novel biological effect of FGD1 in the tumorigenesis of osteosarcoma.

Several studies have revealed that PTEN is a tumor suppressor that modulates tumor cell metabolism, proliferation, and metastasis 28, 29. Dysregulation of PTEN plays a key role in the development of osteosarcoma 10. Exploring the regulatory mechanism of PTEN is essential for better understanding the tumor biology in osteosarcoma. PTEN is reported to be regulated at the transcriptional level by transcription factors, such as TP53, EGFR-1, c-Jun, NF-κB, MKK4 or HES1 30-34. Besides, miRNA, including miRNA-21, miRNA-22, mir-17-92 or mir-367-302b, could bind to the 3'-untranslated region of *PTEN* and suppress its expression in tumor cells 8. Moreover, post-translational modification of PTEN protein, such as phosphorylation, acetylation, and ubiquitination contributes towards regulating the PTEN phosphatase activity, cellular localization, and protein stability 8. Notably, this process is mediated by numerous binding partners of PTEN in tumor cells. In this study, we demonstrated that the N-terminal region of PTEN containing the phosphatase domain interacted with FGD1 in osteosarcoma tumor cells. Further, the study indicated that FGD1 inhibited the phosphatase activity of PTEN and re-activated the PI3K/AKT pathway in cells. Therefore, our results identified FGD1 as a negative regulator of PTEN, which enhances our current understanding of the biology of osteosarcoma.

Traditional cancer therapy, including surgical treatment and chemo-/radio-therapy, has considerably extended the survival time of osteosarcoma patients in the past decades 35. However, serious side effects of chemo-/radio-therapy, acquired resistance to anti-tumor drugs, and refractory features of osteosarcoma after extensive surgical resection make osteosarcoma treatment reach a plateau 35, 36. Recently, immune checkpoint-based therapy has shown promising anti-tumor effects by restoring the immune response in the tumor microenvironment 37. Targeting PD-1 and PD-L1 significantly improves the outcome in various types of cancer, such as melanoma, breast cancer, and non-small cell lung cancer 38-40. It has been well documented that PD-L1 expression is high in approximately 20% of osteosarcoma patients 19, which is consistent with our findings via IHC analysis of osteosarcoma tissue microarray (Figure 5H). Mouse model studies demonstrate that anti-PD-l/anti-PD-L1 treatment markedly reduced the metastasis of osteosarcoma. Meanwhile, pembrolizumab (anti-PD-1 antibody) was found to be effective in treating osteosarcoma patients in a clinical trial (SARC028) 41. Therefore, exploring the regulatory mechanism of PD-1/PD-L1 is important for improving the immunotherapy effect in osteosarcoma. Notably, PD-L1 is reported to be regulated at the transcriptional and post-transcriptional level by various factors. Interestingly, PTEN down-regulates the mRNA levels of PD-L1 in tumor cells 42. This process could be mediated by the PI3K/AKT signaling pathway or NF-κB pathway, which are documented to transcriptionally increase PD-L1 expression in cancer cells. Here, we revealed that FGD1 participated in increasing PD-L1 expression and regulating the tumor immune response in a PTEN-dependent manner. Silencing FGD1 improved the anti-PD-1 immunotherapy effect on osteosarcoma in mice. Therefore, targeting FGD1 might provide a direction in enhancing the anti-PD-1 based immunotherapeutic efficacy in the treatment of osteosarcoma in future experimental and clinical trials.

In conclusion, our study revealed that FGD1 was aberrantly overexpressed in osteosarcoma specimens and associated with unfavorable prognosis. FGD1 contributed to osteosarcoma tumor cell proliferation and invasion *in vivo* and *in vitro* (Figure 6H). Moreover, FGD1 interacted with PTEN to inhibit its phosphatase activity and re-activated PI3K/AKT/ NF-κB pathway in osteosarcoma tumor cells (Figure 6H). Importantly, FGD1 was proved to increase PD-L1 expression and modulated immune response in a PTEN-dependent manner (Figure 6H). Collectively, FGD1 might be a potential target for improving the survival rate of patients with osteosarcoma.

## Supplementary Material

Supplementary methods, figures, and tables.

## Abbreviations

FGD1: FYVE, RhoGEF and PH domain-containing protein 1
PTEN: Phosphatase and tensin homolog
PI3K: phosphatidylinositol 3-kinase
PD-1: programmed cell death receptor-1
IP: immunoprecipitated
