# Supplementary material for: FGD1 promotes tumor progression and regulates tumor immune response in osteosarcoma via inhibiting PTEN activity
Source: Theranostics. 2020 Feb 3;10(6):2859–71. doi: 10.7150/thno.41279 (PMC7052884; doi:10.7150/thno.41279)
Supplement: Supplementary file 1 — Supplementary methods, figures, and tables. [file thnov10p2859s1.pdf]

FGD1 promotes tumour progression and regulates tumour immune response in osteosarcoma via inhibiting PTEN activity

Wei Wu, Doudou Jing, Binwu Hu, Binlong Zhong , Xiangyu Deng, Xin Jin, Zengwu Shao

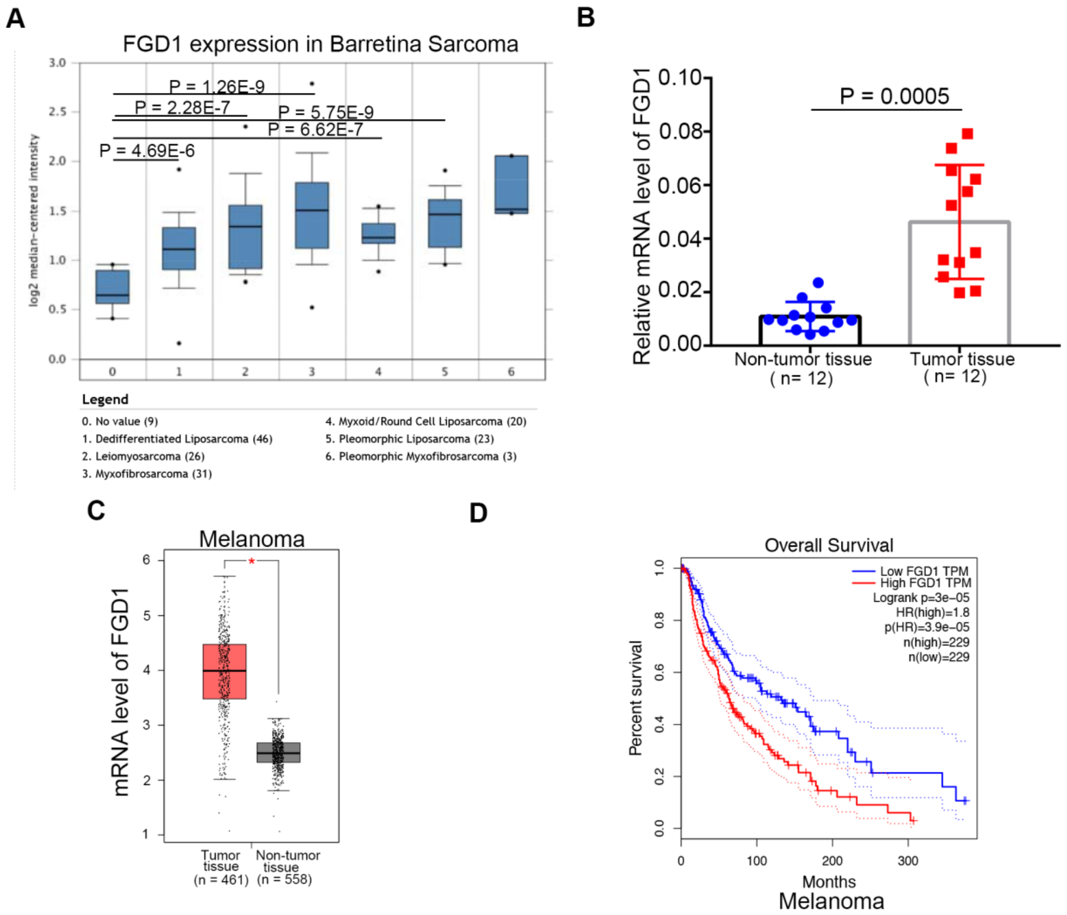

Figure S1. A, the mRNA expression levels of FGD1 in Sarcoma by using Oncomine data set. **B**, the mRNA expression levels of FGD1 in osteosarcoma tumor tissue and adjacent non-tumor tissues (n = 12). **C-D**, the mRNA expression levels of FGD1 and survival rate of patients with different expression levels of FGD1 in melanoma analyzed by the GEPIA web tool.

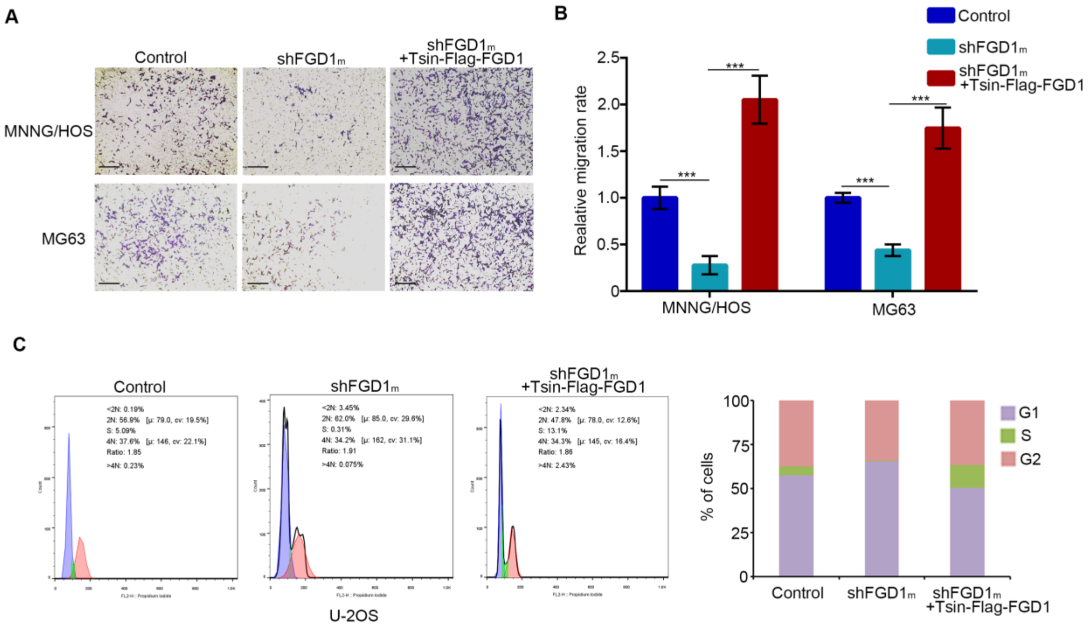

Figure S2 .**A-B**, MNNG/HOS and MG63 cells were transfected with indicated constructs. After 72 h transfection, cells were harvested for transwell assay. Data presented as Mean ± SD with three replicates. ***, P < 0.001. **C**, U-2OS cells were transfected with indicated constructs. 72 h post-transfection, cells were subjected to cell cycle analysis.

A

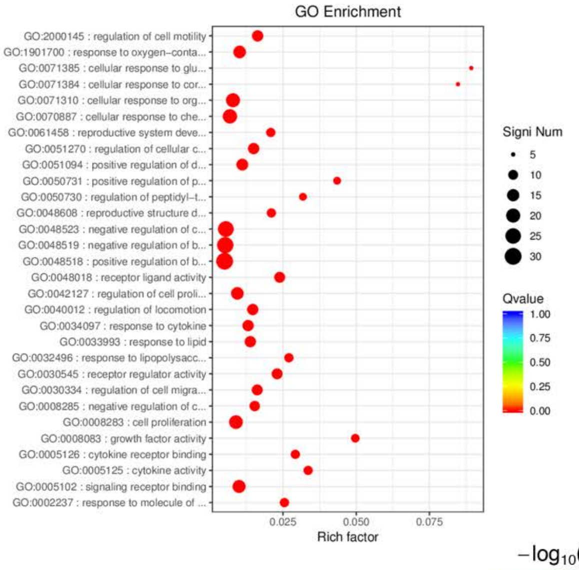

B

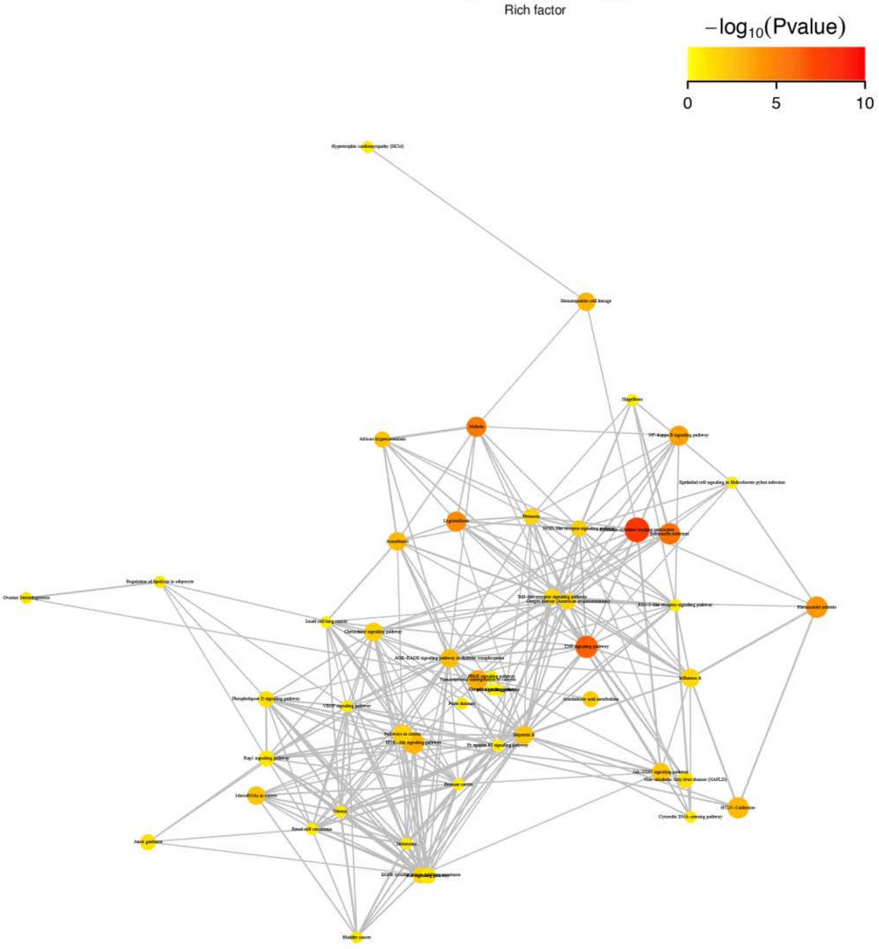

Figure S3. A-B，GO enrichment (a) and KEGG enrichment (b) pathway analysis of the RNA-seq data set after silencing FGD1 in MNNG/HOS cells.

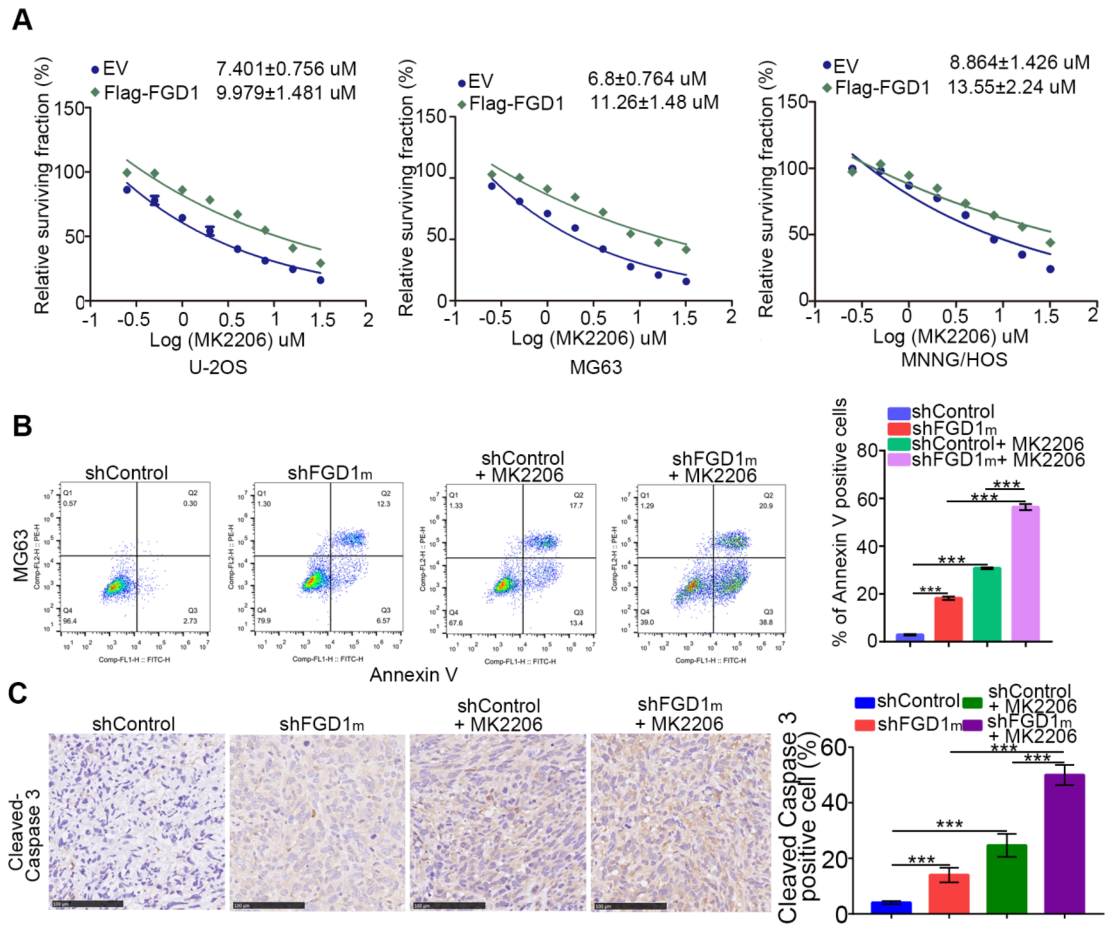

Figure S4. A, detecting the IC50 values after transfected with indicated plasmids in U-2OS, MG63 and MNNG/HOS cells. **B**, MG63 cells were infected with indicated shRNAs. After 72 h, cells were treat with or without MK2206 (10uM) for other 24 h and cells were harvested for Annexin V/PE analysis. **C**, the xenografts tumor were harvested for cleaved-caspase 3 staining. Data presented as Mean ± SD with three replicates. ***, $P < 0.001$.

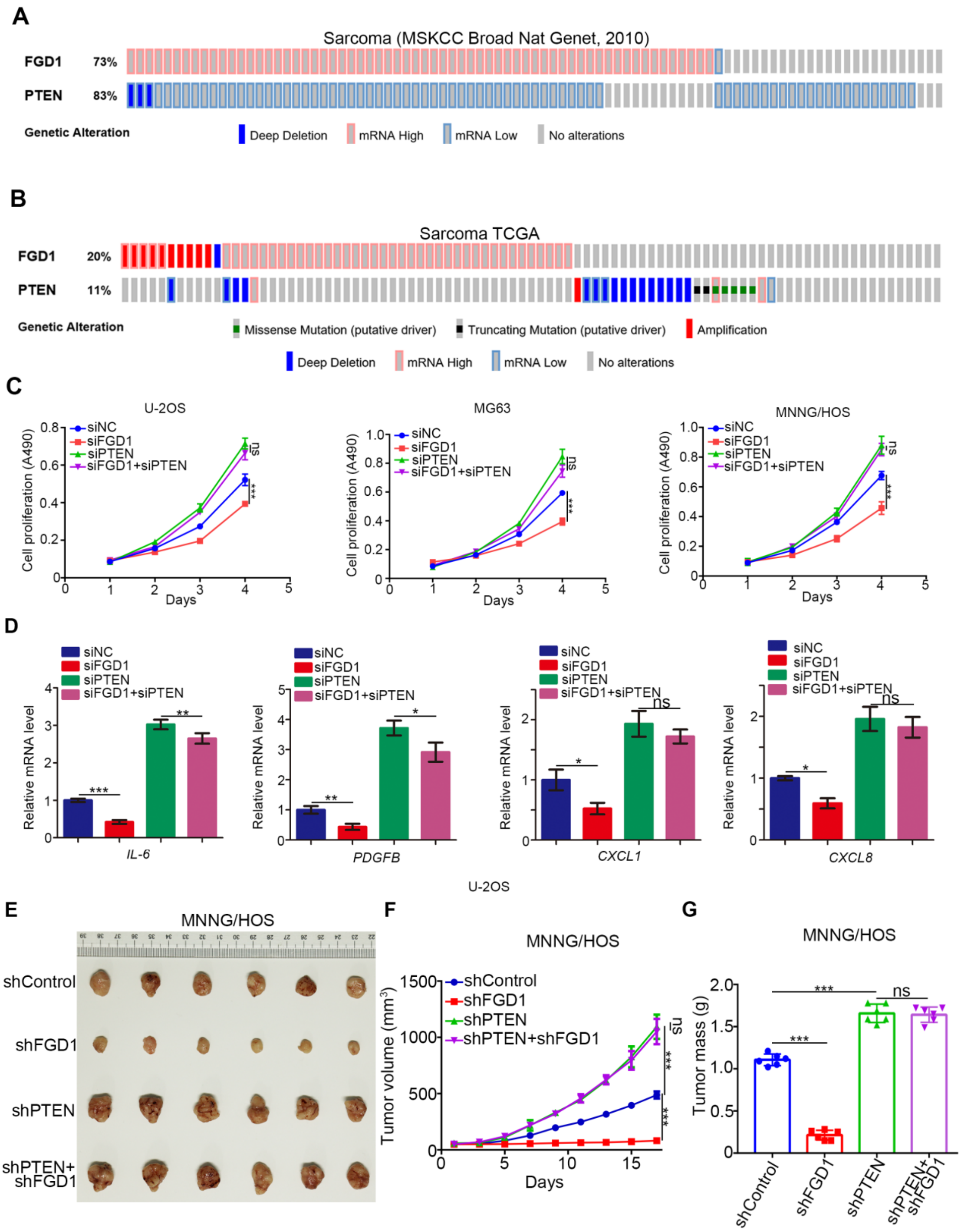

Figure S5. A and B, the mRNA expression level of point mutation of FGD1 or PTEN in two different sarcoma data sets. **C and D**, U-2OS, MG63 and MNNG/HOS cells were transfected with indicated siRNAs. 48 h post-transfection, cells were subjected to MTS assay (c).and RT-qPCR analysis. Data presented as Mean ± SD with three replicates. n.s., not significant; *, $P < 0.05$; **, $P < 0.01$; ***, $P < 0.001$. **E-G**，MNNG/HOS cells were transfected with indicated constructs. After

72 h transfection and puromycine selection, cells were injected subcutaneously into the nude mice for xenografts assay (E). The tumor growth curve (F) and excised tumor mass (G) as indicated. ns，not significant; ***, $P < 0.001$.

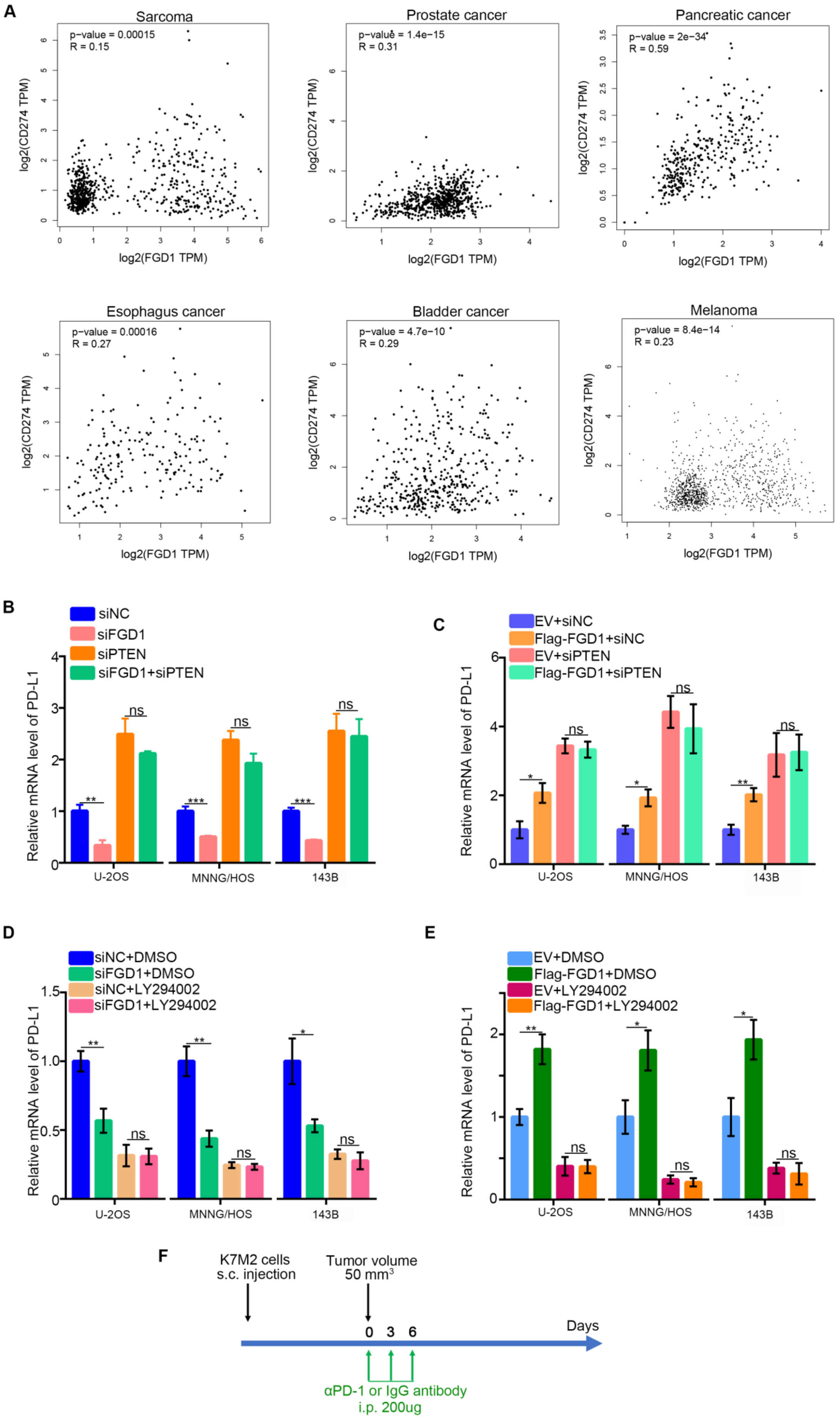
A
Sarcoma
p−value = 0.00015
R = 0.15
Prostate cancer
p−value = 1.4e−15
R = 0.31
Pancreatic cancer
p−value = 2e−34
R = 0.59
Esophagus cancer
p−value = 0.00016
R = 0.27
Bladder cancer
p−value = 4.7e−10
R = 0.29
Melanoma
p−value = 8.4e−14
R = 0.23
log2(CD274 TPM)
log2(FGD1 TPM)
B
siNC
siFGD1
siPTEN
siFGD1+siPTEN
Relative mRNA level of PD-L1
U-2OS
MNNG/HOS
143B
C
EV+siNC
Flag-FGD1+siNC
EV+siPTEN
Flag-FGD1+siPTEN
D
siNC+DMSO
siFGD1+DMSO
siNC+LY294002
siFGD1+LY294002
E
EV+DMSO
Flag-FGD1+DMSO
EV+LY294002
Flag-FGD1+LY294002
F
K7M2 cells
s.c. injection
Tumor volume
50 mm3
Days
αPD-1 or IgG antibody
i.p. 200ug

Figure S6. A, the correlation between FGD1 and CD274 (PD-L1) in different types of cancer were analyzed by the GEPIA web tool. **B**, U-2OS, MNNG/HOS and 143b cells were infected with indicated siRNAs. After 48 h, cells were harvested for RT-qPCR analysis. Data presented as Mean ± SD with three replicates. n.s., not significant; **, P < 0.01; ***, P < 0.001. **b**, U-2OS, MNNG/HOS and 143b cells were transfected with indicated constructs. 48 h post-transfection, cells were subjected to RT-qPCR analysis. Data presented as Mean ± SD with three replicates. n.s., not significant; *, P < 0.05; **, P < 0.01. **C**, U-2OS, MNNG/HOS and 143B cells were infected with indicated siRNAs. After 48 h, cells were treated with or without LY294002 (40 uM) for other 24 h. WCL of cells collected for RT-qPCR analysis. Data presented as Mean ± SD with three replicates. n.s., not significant; *, P < 0.05; **, P < 0.01. **D**, U-2OS, MNNG/HOS and 143B cells were infected with indicated constructs. After 48 h, cells were treated with or without LY294002 (40 uM) for other 24 h. WCL of cells collected for RT-qPCR analysis. Data presented as Mean ± SD with three replicates. n.s., not significant; *, P < 0.05; **, P < 0.01.**E**, U-2OS, MNNG/HOS and 143B cells were transfected with indicated constructs. After 48 h, cells were treated with or without LY294002 (40 uM) for other 24 h. WCL of cells collected for RT-qPCR analysis. Data presented as Mean ± SD with three replicates. n.s., not significant; *, P < 0.05; **, P < 0.01. **F**, K7M2 cells were infected with lentivirus vectors expressing control or Fgd1-specific shRNAs. 72h after puromycin selection, 5 x 10^6 cells were injected subcutaneously into C57BL/6 mice. Mice (n=5/group) were treated with anti-PD-L1 (200 µg) or non-specific IgG for 45 days.

Supplementary Methods

Correlation analysis using the GEPIA web tool

The online database Gene Expression Profiling Interactive Analysis (GEPIA, http://gepia.cancerpku.cn/index.html) was used to analyze the RNA sequencing expression data. GEPIA performs a pairwise gene correlation analysis for any given set expression data using Pearson correlation statistics.

Cell proliferation assay

The ability of cell proliferation was tested by MTS(3-(4,5dimethylthiazol-2-yl)-5-(3-carboxymethoxyphenyl)-2-(4sulfophenyl)-2H-tetrazolium, inner salt) assay. MNNG/HOS,MG-63 and U2-OS cells were plated at a density of 2×103 cells per well with 100ul α-MEM medium in 96-well for 24 hours. According to the assumed concentration gradient, several small molecule inhibitors were added to the medium. After 72 hours, each well added to 20ul MTS and incubated for 1 hour in the humidified incubator in the dark. The absorbance at 490 nm was detected with a spectrophotometer.

Real-time RT-PCR

After the cells or tissues in each group were treated, the total RNA was extracted from the cells or tissues by using TRIzol reagent (Thermo Fisher Scientific, USA). Using the cDNA Reverse Transcription kit (PrimeScript™ RT reagent Kit, Code No. RR037A ,Takara, Dalian, China), the total RNA was reverse transcribed to cDNA in a final volume of 20μL with random primers under standard conditions, and real-time PCR analysis were carried out using a PCR kit (TB Green™

Fast qPCR Mix, Code No. RR430A, Takara, Dalian, China) according to the manufacturer's instructions. All the values were normalized to GAPDH, and the data were collected and using the $2^{-\triangle\triangle CT}$ method to analyze. The sequences of RT-qPCR primers as indicated in Table S1.

Tissue microarray and immunohistochemistry (IHC)

The tissue microarray slides were purchased from US Biomax (OS804c). The tissue microarray specimens were immunostained with FGD1 (Thermofisher, PA5-40416, dilution 1: 50) and PD-L1 antibodies (Cell Signaling Technology, 13684S, dilution 1:50). Staining intensity was scored in a blinded fashion: 1 = weak staining at 100× magnification but little or no staining at 40× magnification; 2 = medium staining at 40× magnification; 3 = strong staining at 40× magnification [15]. The degree of immunostaining was scored by two independent pathologists who were blinded to the clinical details. The IHC scores were calculated by the percentage of positive cells x the staining intensity.

Generation and treatment of K7M2 xenografts in mice

The 6-week-old C57BL/6 mice purchased from Charles River Laboratories (Wuhan, China). All the animal experimental procedures were approved by the Ethics Committee of Tongji Medical College, Huazhong University of Science and Technology. K7M2 cells (5×10^6 in 100 µl 1×PBS) infected with lentivirus of shControl or shFgd1 were injected s.c. into the right flank of mice. The volume of xenografts was measured every other day and calculated using the formula $LxW^2x0.5$. After xenografts reached a size of approximately 50 mm^3, mice carrying similar type of tumors were randomized into different groups and treated with anti-PD-1 (BioXcell, Clone RMP1-14) or

IgG (BioXcell, Clone 2A3) (200 μg, i.p., given at days 0, 3, 6). Mice were euthanized and tumors collected from all animals once tumors reached a volume of 300 mm^3.

For flow cytometry analysis of mouse tissue samples, tumors were cut into small pieces and digested with 2 mg/ml collagenase (Sigma, USA) in DMEM for 1 h at 37°C. Cells were filtered through 70 μm nylon strainer and re-suspended in red blood cell lysis buffer (Biolegend) for 3 min at room temperature. Cells were then suspended in PBS with 2% BSA and co-stained with the following antibodies: CD45 (Biolegend, 103112, APC conjugated), CD4 (Biolegend, 100510, FITC conjugated), CD8 (Biolegend, 100708, PE conjugated) , CD11b (Biolegend ,101212, APC conjugated), Gr1 ((Biolegend, 108406, FITC conjugated)). After incubated with antibody for 15 min, cells were washed with PBS and analyzed on flow cytometer.

In vitro binding assay

Flag-FGD1, Myc-PTEN-N and Myc-PTEN-C protein were translated in vitro following the manufacture protocol of TNT ® Quick coupled Translation System Technical (Promega, #TM045). The in vitro translated FGD1 and the recombination protein PTEN-N and PTEN-C were mixed for immunoprecipitation assay. Then, the reaction and samples were sent for western blotting analysis.

Table S1: Sequences of RT-qPCR primers

**Species**	**Gene**	**Forward (5'-3')**	**Reverse (5'-3')**
Human	*GAPDH*	CCAGAACATCATCCCTGCCT	CCTGCTTCACCACCTTCTTG
Human	*PD-L1*	GGTGCCGACTACAAGCGAAT	AGCCCTCAGCCTGACATGTC

Human	*FGD1*	AAAATGAACCCTTGGTGCTG	GGCTGAAGTACCAGCTGAGG
Human	*IL-6*	TACCCCCAGGAGAAGATTCC	TTTTCTGCCAGTGCCTCTTT
Human	*PDGFB*	CTTTAAGAAGGCCACGGTGA	CTAGGCTCCAAGGGTCTCCT
Human	*CXCL1*	AGGGAATTCACCCCAAGAAC	TGGATTTGTCACTGTTCAGCA
Human	*CXCL8*	GTGCAGTTTTGCCAAGGAGT	CTCTGCACCCAGTTTTCCTT
Mouse	*Gapdh*	AGGTTGTCTCCTGCGACTTCA	GGGTGGTCCAGGGTTTCTTACT
Mouse	*Pd-l1*	AATGCTGCCCTTCAGATCAC	ATAACCCTCGGCCTGACATA

Table S2: Sequences of gene-specific shRNAs

shFGD1-1	5′- CCGGCCTGTGATTGTCGCCTCGGATCTCGAGATCCGAGGCGACAATCACAGGTTTTTG-3′
shFGD1-2	5′- CCGGGCCCTTCAATTCTATCACCAACTCGAGTTGGTGATAGAATTGAAGGGCTTTTTG-3′
shFgd1-1	5′-CCGGCCGTGTTGAATATTCATCTAACTCGAGTTAGATGAATATTCAACACGGTTTTTG-3′
shFgd1-2	5′-CCGGGAGCGGTCTACCCAGTTTAAACTCGAGTTTAAACTGGGTAGACCGCTCTTTTTG-3′
